# Closure of Interatrial Septal Communications: Adverse Events and Lessons Learned

**DOI:** 10.4021/cr17w

**Published:** 2011-01-20

**Authors:** Philipp Wagdi

**Affiliations:** HerzZentrum Hirslanden, Witellikerstrasse 36, 8008 Zurich, Switzerland. Email: wagdi@herzzentrum.ch

**Keywords:** Adverse events, Classification, Percutaneous closure, Patent foramen ovale, Atrial septal defect

## Abstract

**Background:**

Percutaneous closure of interatrial septal communications (IASC) is generally being regarded as a safe and straightforward intervention. Reporting and classification of adverse events (AE) as is the case for percutaneous coronary intervention (PCI) is not standardized. Also, the focus of reported larger studies has not been primarily on AE and strategies to avoid them.

**Methods:**

The data of all 112 consecutive patients undergoing IASC by a single operator were reviewed. In analogy to classification for PCI, an AE was considered to be major if any of the following occurred: death, major or minor stroke, myocardial infarction, the need for an originally unplanned additional surgery or intervention or blood transfusion. Every AE and how it may have been avoided is reviewed in detail.

**Results:**

Major AE according to the suggested classification occurred in 2.7% of patients, including tamponade in 1 patient necessitating thoracotomy 7 months after IASC, percutaneous retrieval of an embolized device in 1 patient, and ambulatory same day surgical treatment of an arteriovenous fistula in 1 patient.

**Conclusions:**

The proposed new classification of AE provides a unified and comparable approach for IASC procedures. Retrospectively, two of the 3 major AE could have probably been avoided by more thoughtful patient and material selection.

## Introduction

Percutaneous closure (C) of interatrial septal communications (IASC) has gained widespread acceptance, generally being regarded as a safe and straightforward intervention [1, 2]. The indication for closure of patent foramen ovale (PFO) is still controversial and ranges from being almost ubiquitous to rather reserved [3, 4]. Information about possible major or minor, early or late peri-interventional complications is less widespread. This may be due to the fact that adverse events (AE) are rather rare, that minor ones may not be reported at all, while major complications are usually published in specialized interventional journals as case reports and are thus less accessible to a broad public. Although complications have been reported in larger studies, the focus may not be primarily on AE [1, 2]. Also, whereas classification of severity of AE has been largely standardized, for example for percutaneous coronary interventions (PCI) [5], there is to our knowledge no such universally followed practice for IASC-C, maybe because some additional issues have to be considered (for example device dislodgement, etc.) for the latter intervention. We therefore sought to review the data of all consecutive patients who underwent an IASC-C by a single operator between January 2006 and December 2010, compiling all major and minor, early and late adverse effects observed during this period. We also sought to apply an adapted classification of AE and we analyzed whether the encountered AE could have been avoided. In the light of our findings, we examine the validity of our approach to IASC-C hitherto.

## Methods

From January 2006 to December 2010, 112 patients underwent IASC-C. Patient characteristics are presented in Table 1. All patients were seen at our institute at least one month and six months after the procedure for transthoracic (TTE) and transoesophageal (TEE) echocardiography. No patients were lost to follow up, and all patients were systematically interviewed in addition to the echocardiographic exam. In addition, all patients were advised to contact us immediately if any symptoms whatsoever occurred and such patients were seen in between and after these intervals. No AE, other than those listed below, were reported by either the treating physician or external centre. Many patients were seen thereafter periodically mainly for other issues (control of blood pressure, stress test, etc.).

**Table 1:** Patient Characteristics

Patients	N = 112
Age in years	55.2 ± 14.9 (21 - 83)
Gender	55 males, 57 females
Months since implantation	22.4 ± 13.4 (1 - 60)
Type of IASC	112 PFO, 14 ASD
Type of device implanted	A = 46, F = 21, C = 23, S = 14, P = 8

ASD: atrial septal defect

IASC: interatrial septal communication

PFO: patent foramen ovale

Device: A = Amplatzer®, C = Atriasept Cardia®, F= Occlutec Figulla®, P = Premere®, S = Swissimplant Solysafe®

AE were classified into major or minor according to the following criteria. An AE was considered to be major if any of the following occurred: death, major or minor stroke, myocardial infarction, the need for an originally unplanned additional surgical or percutaneous intervention or the need for blood transfusion. A complication of intermediate severity was considered to be present if it necessitated the additional intake of originally unplanned medication for more than three months (for example oral anticoagulation or Amiodarone), or more than three unplanned ambulatory consultations or short hospitalizations related to the procedure or the patient’s perception of it. All other procedure-related events were considered to be minor.

## Results

### Late tamponade and thoracotomy (n = 1)

**Figure 1:**
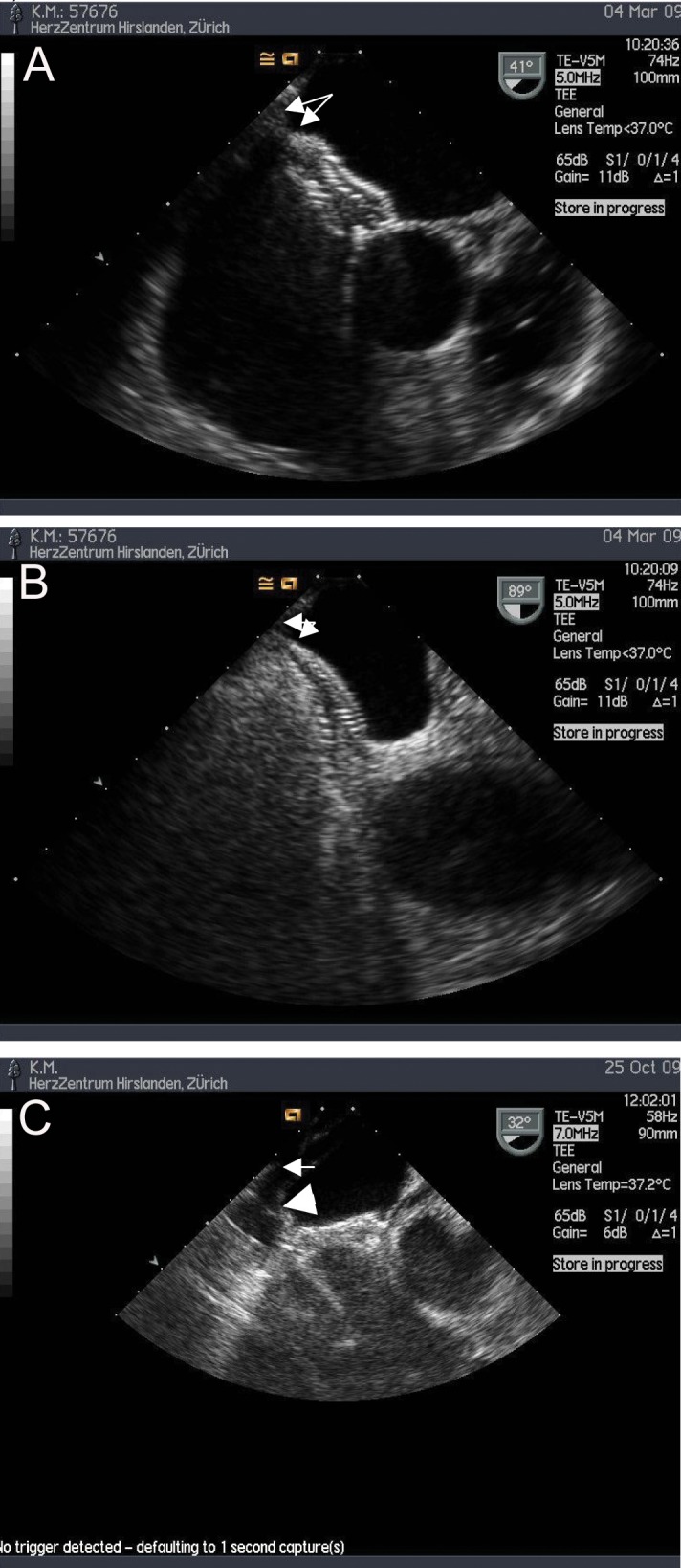
(A) and (B) Intra-interventional TEE showing respective positions of device edges and atrial wall (arrows). (C) Large arrowhead showing ruptured left atrial wall, small arrow showing pericardium.

A 65-year-old female patient had undergone repeated extensive abdominal surgery for recurrent diverticulitis, bowel perforation and ileus. She had recurrently complained of shortness of breath and chest pain, and bouts of cyanosis and atrial fibrillation had been documented. Coronary artery disease and relevant valvular disease were ruled out, echocardiography documented a large atrial septal defect (ASD) with bidirectional shunting, biatrial dilatation and moderate pulmonary arterial hypertension (systolic pulmonary artery pressure 45 mmHg). Balloon measurement yielded a diameter of 19 mm, after which the defect was uneventfully closed with a 21 mm Occlutec Figulla® device (Fig. 1A, B). Clinical and echocardiographic controls after one month and four months were unrevealing, except for an episode of atrial fibrillation treated with Amiodarone and oral anticoagulation for two months, during which sinus rhythm was restored and both drugs were then stopped, the patient taking only 100 mg of acetyl salicylic acid (ASA). Seven months after the intervention, the patient presented with acute chest pain, dizziness, shortness of breath and hypotension. Cardiac tamponade was diagnosed by TTE and TEE (Fig. 1C) and the patient underwent thoracotomy. The surgeon found 300 ml of partially coagulated haemorrhagic pericardial effusion and two tears of about 3 - 4 mm in the atrial roof on both sides. A pericardial patch with U-shaped Teflon-felt supported sutures was used to seal the defects. Unusual tissue friability was noted, also during the excision of the left atrial appendage, requiring consolidation sutures and lengthy haemostasis. The surgeon decided to leave the device in situ because the extensive healing reaction on the device would have necessitated major reconstruction of the atria if the device had been removed. Postoperative recovery was complicated by a subacute sternal infection after four weeks requiring re-sternotomy and debridement, as well as prolonged antibiotic treatment. The patient was seen four months after the last operation and was oligosymptomatic but for osteopathic pain.

### Device embolization and successful percutaneous retrieval (n = 1)

In one patient, late device dislodgement was documented and the device was retrieved uneventfully as reported previously [6].

### Arteriovenous fistula (n = 1)

A 38-year-old male patient had undergone uneventful closure of a PFO with a 20 mm Solysafe® device. Two weeks later, he returned with a painful pulsating swelling at the puncture site, an arteriovenous (AV) fistula was diagnosed and treated surgically on a same-day ambulatory basis, the surgeon described a connection between the femoral vein and a side branch of the superficial femoral artery crossing the vein. Recovery was uneventful.

### Thrombus formation on an ASD device (n = 1)

**Figure 2:**
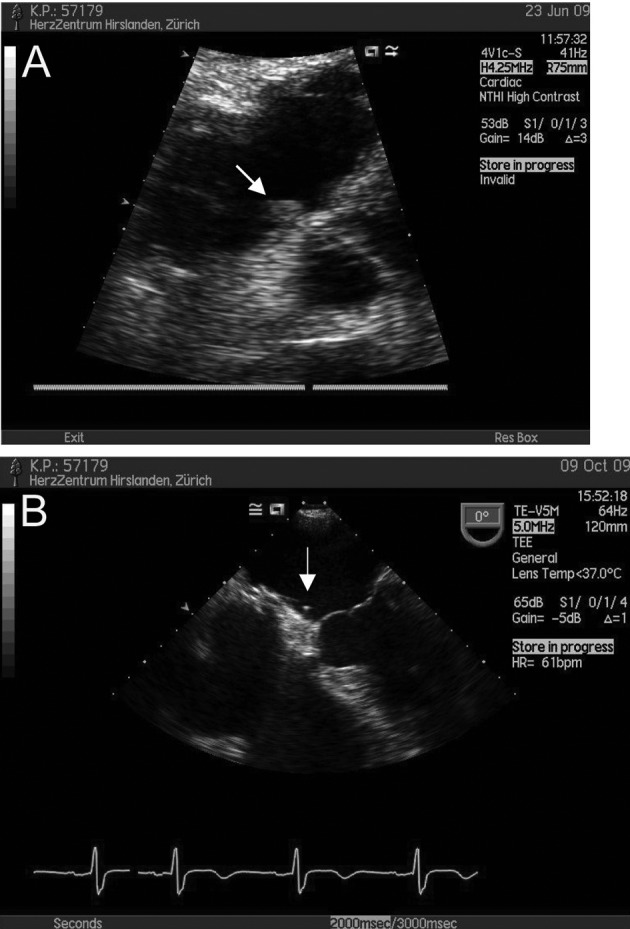
(A) TTE showing a thrombus on the right atrial side of the device (arrow). (B) TTE 4 months later, showing a thread-like structure attached to the left sided disc.

A 57-year-old patient with insulin-dependent diabetes and recurrent septic bouts due to infected Charcot-Marie-Tooth disease suffered recurrent pulmonary oedema, atrial fibrillation and troponin-positive chest pain. Diagnostic work up showed significant coronary artery disease with critical left anterior descending artery (LAD) stenosis, a large ASD and draining of the left upper pulmonary into the azygos vein. Pulmonary vascular surgery for the latter condition was declined and it was decided to perform PCI and ASD closure. One month after uneventful PCI of the LAD and closure of the 32 mm ASD with a 32 mm Atriasept Cardia® device, cardioversion was performed. On follow up, a large sessile right atrial thrombus was seen in TTE (Fig. 2A). At that time the patient was on low molecular weight heparin (LMWH), ASA and Clopidogrel medication. Before initiating oral anticoagulation, a coagulopathy-screening was performed as detailed previously [7], but none was detected. Warfarin was added and LMWH discontinued after INR reached 2.5. Two months later and under documented adequate compliance, TEE showed resolution of the right atrial thrombus, but development of a thread-like left atrial (thrombotic) structure (Fig. 2B). Oral anticoagulation was continued with ASA and Clopidogrel stopped. Two months later, TEE documented resolution of all thrombotic material, since then the patient has been symptom- and event-free on ASA alone.

### Atrial fibrillation and conversion to sinus rhythm (n = 9), radiofrequency ablation and left atrial appendage closure (n = 1)

**Figure 3:**
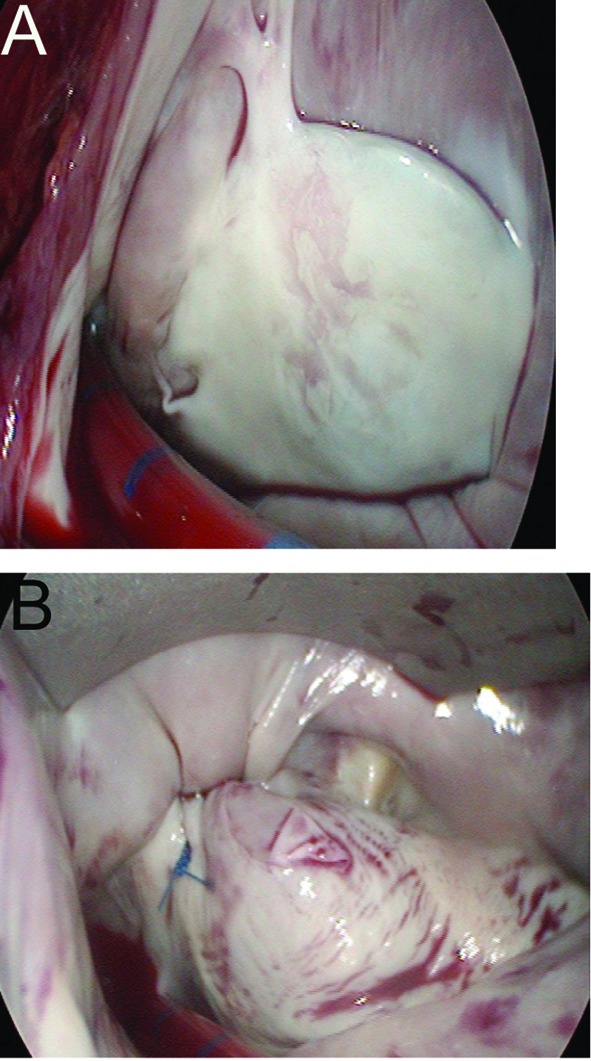
(A) Endothelialized left atrial surface of the PFO closure device. (B) Appearance of the ligated left atrial appendage.

Of the 112 patients examined, 10 (9%) developed atrial fibrillation and all of them reverted to sinus rhythm within less than two months except for one patient who declined cardioversion and Amiodarone medication, being asymptomatic under beta-blocker therapy. One year after PFO closure, she experienced repeated bleeding episodes under oral anticoagulation. She underwent uneventful videoscopic minimal-invasive surgery (Fig. 3) with ligature of the left atrial appendage, as well as biatrial radiofrequency ablation. Resting ECG and 7-day ECG showed stable atrial rhythm, with no episodes of atrial fibrillation or any other dysrhythmia after oral anticoagulation had been stopped. The detailed findings of our patient cohort developing atrial fibrillation after IASC-C have already been reported and discussed [8].

### Transient ST elevation (air embolism) with no myocardial damage (n = 1)

**Figure 4:**
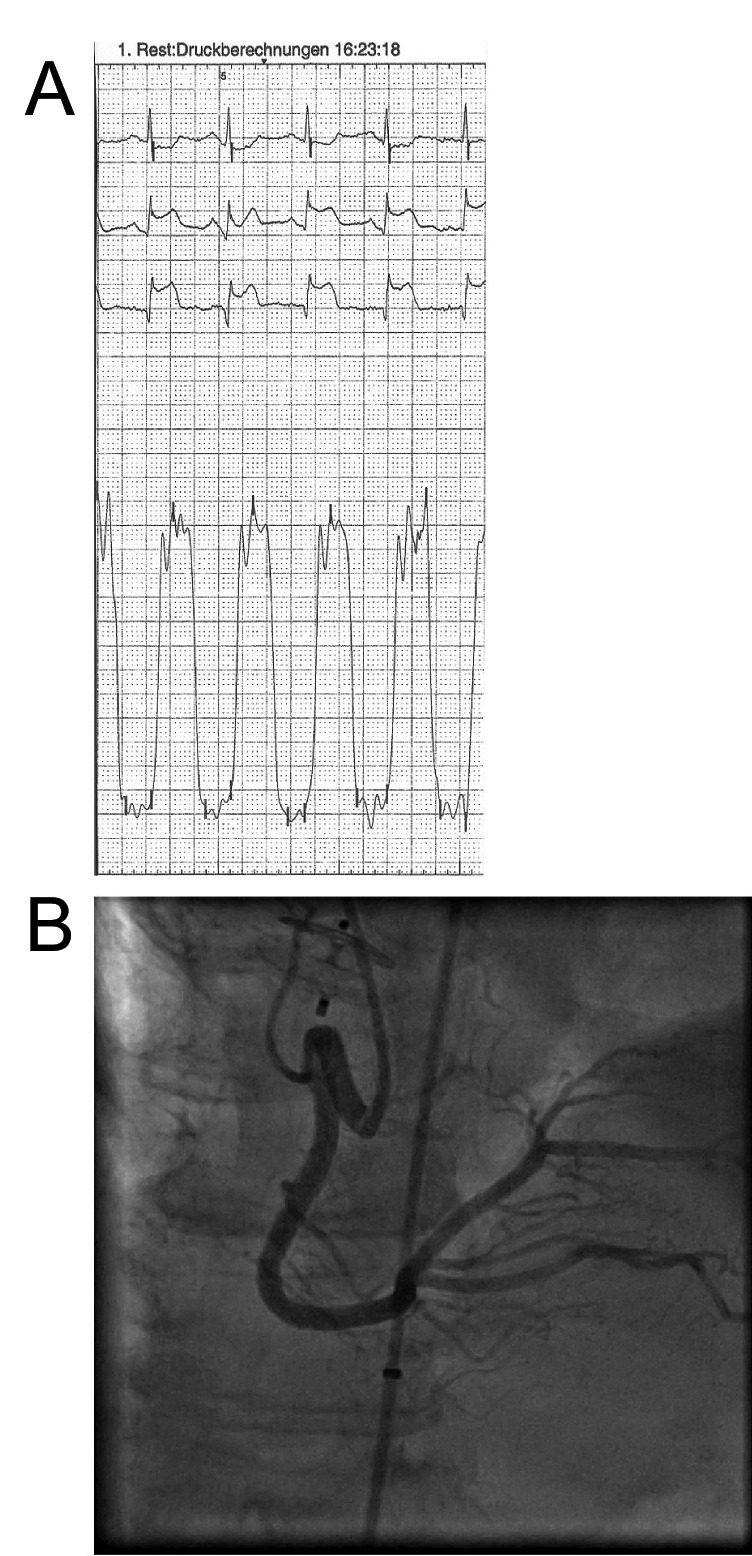
(A) Tracing showing ST-segment elevation in inferior leads after device placement due to air embolism in the right coronary artery. (B) Immediate coronary angiography showing resolution of the air bubbles and patent right (and left (not shown here)) coronary artery.

In a 69-year-old female patient, a transient massive ST-segment elevation (Fig. 4A) in the inferior leads occurred during device deployment for a PFO. Air embolism was suspected and immediate coronary angiography (CA) was performed within 2 minutes with a guiding catheter, an aspiration device being at hand for aspiration of a potential, vessel-occluding, air bubble. This proved unnecessary, contrast injection showed no air bubbles and the ST-elevation resolved spontaneously (Fig. 4B). Troponin I levels measured six and twelve hours after the intervention peaked at 1 µg/L (normal < 0.1 µg/L). CK and CK-MB levels did not rise, the ECG was unchanged with no signs of acute or subacute ischaemia. A cerebral MRI showed no signs of ischaemia or sequelae of air embolism.

### Partially successful or unsuccessful interventions, “redos”

**Figure 5:**
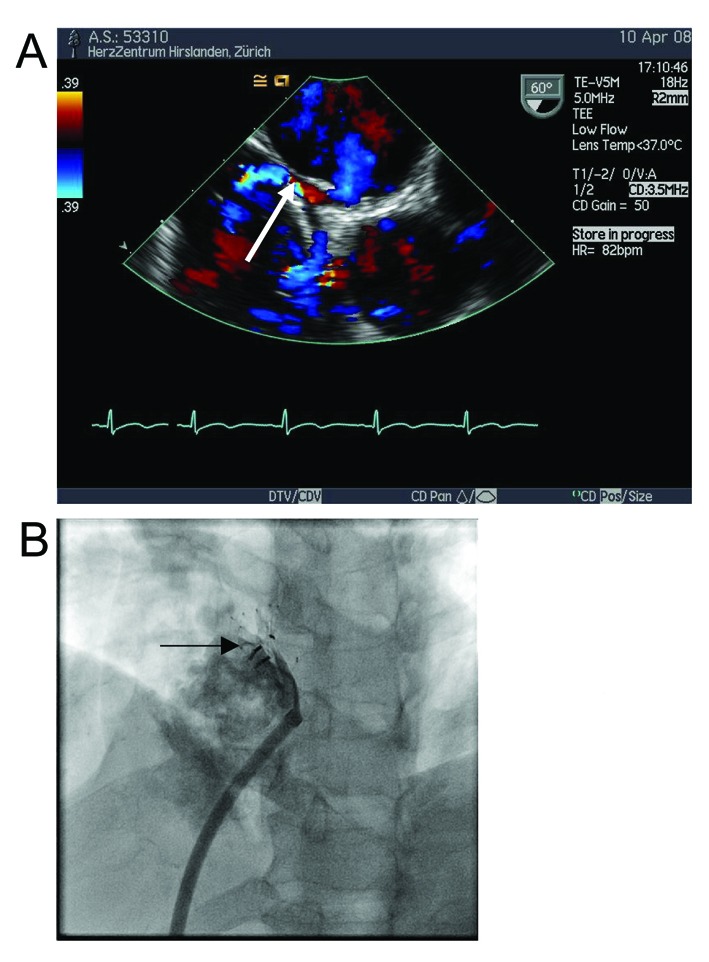
(A) Follow up TEE of the patient with central residual shunt (white arrow) following PFO closure with a first Premere® device. (B) Implantation of a second device (black arrow pointing at knob of second device) demonstrating tight closure.

**Figure 6:**
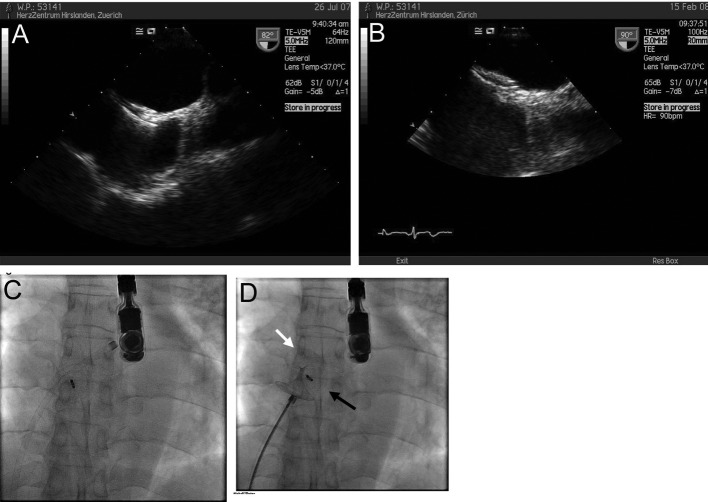
(A) Intraprocedural TEE showing adequate position of both device arms. (B) Follow up TEE showing leftward malposition of the right atrial disc. (C) Original Amplatzer® device in place, with introducer passed through the residual defect. (D) Second Amplatzer® device in place (white arrow), with tightly closed residual shunt (not shown), with both device ends standing out perpendicularly to the first device (black arrow) and into the respective atrial cavity.

**Figure 7:**
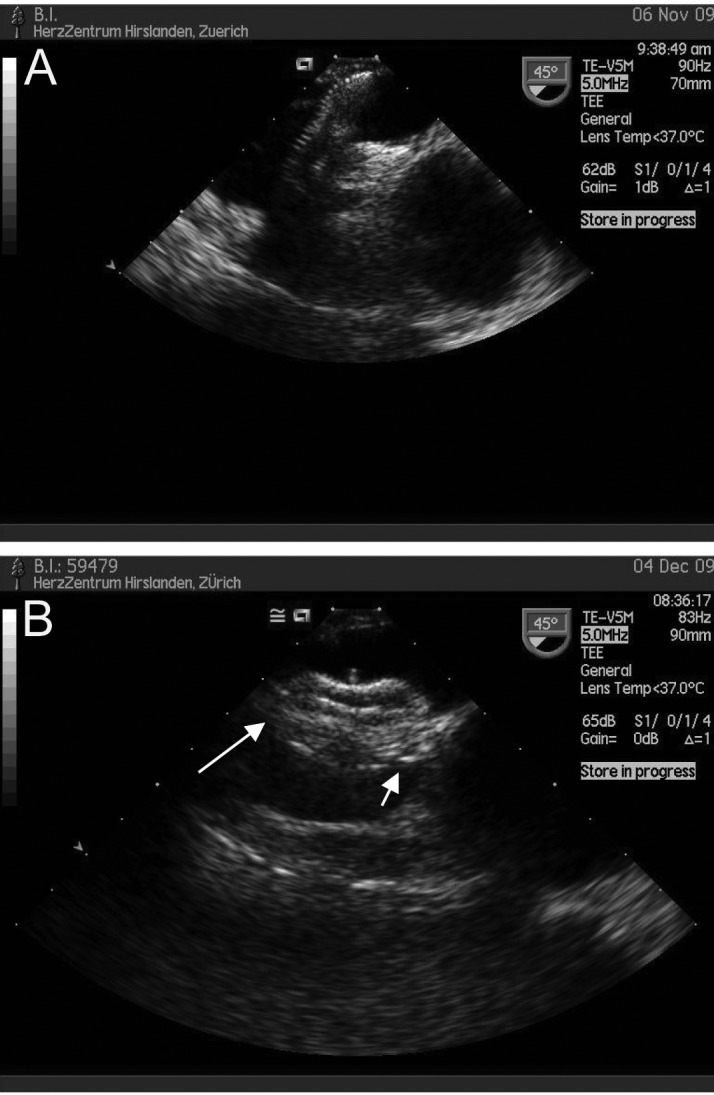
(A) 35 mm PFO device in place swung around by the large atrial septal aneurysm. (B) Stable 18 mm ASD device filling the defect, grasping both septum primum and secundum (arrows), and not allowing any rocking motion by the atrial septal aneurysm anymore.

1) In three patients, a rest-shunt was documented after initial uneventful PFO closure; no balloon measurement of the foramen had been made in any of the three during the intervention and dimensions measured by TEE were relied on. In the first patient, a 37-year-old male, TEE control 6 months after PFO closure documented a large residual shunt (Fig. 5A). A second Premere® device was placed (Fig. 5B), without residual shunt with good device position, as documented by TEE six months later. The second patient, a 44-year-old male, initially had a 25 mm Amplatzer® device placed. Intrainterventional TEE documented adequate device position (Fig. 6A). Follow up showed displacement of the aortic portion of the right atrial disc to the left side (Fig. 6B) and a residual shunt. After crossing the residual defect, a second Amplatzer® device was inserted, but both on TEE and on fluoroscopy (Fig. 6C) was seen to ride on the first device perpendicularly, both discs protruding into the atrial lumen. It was then decided to retrieve the device. On follow up we suggested to the patient that an attempt using a less bulky device should be made, which he declined. The third patient decided to undergo a second closure attempt only if neurologic symptomatology recurred. All three patients are on 100 mg ASA, with no AE occurring since the last intervention.

2) A 69-year-old female was diagnosed as having a large PFO, an aneurysm of the interatrial septum, a large Chiari network and Eustachian valve. A stretched defect diameter of 18 mm was measured and a cribriform 35 mm Amplatzer® device inserted. Because a large amplitude movement of the aneurysmatic atrial septum persisted inspite of the device and the latter was almost impaling the aortic root (Fig. 7A), it was decided to retrieve the device and to try implanting an 18 mm ASD device. No such one was available, so the intervention had to be postponed. The device was successfully implanted one month later (Fig. 7B) and recovery was uneventful.

## Discussion

In this series, 3 major AE (2.7%), 1 intermediate severity AE (0.09%) and 11 minor AE (10%) occurred.

### Major AE

The most dire complication in our series resulted from late perforation of the atrial roof by the device, resulting in tamponade and necessitating thoracotomy and patch repair. Although we had used angiographic balloon sizing of the defect (the intervention temporally occurring after the lessons learned from cases in which we did not balloon size), the problem lay in the fact that potential compromise of neighbouring cardiac structures was not recognized by TEE. In addition, unusual tissue friability as reported by the surgeon may have played a role in facilitating perforation.

As pointed out earlier, undersizing may have contributed to device dislocation [6]. The only AV fistula resulted from the inadvertent puncture, as described by the surgeon, of an arterial side branch, not of the common or superficial femoral artery and this was not really avoidable in our judgement. Also, no spurious aneurysms occurred in our series, this may be explained by the fact that we “volume-preloaded” the venous side with 500 ml of colloid infusion prior to venous puncture, and asked the patient to perform the Valsalva manoeuvre during it.

### AE of intermediate severity

Having excluded thrombophilia, thrombus formation in our case occurred despite the patient receiving a therapeutic dose of LMWH, ASA and Clopidogrel. Replacing LMWH by Warfarin resulted in resolution of the right-sided thrombus, but did not prevent formation of a small left-sided one. Fortunately no neurological event occurred, hopefully due to tight follow up (including securing optimal INR levels) and vigorous treatment. The reason for the formation of the left sided thrombus remains unclear. It may be that “controlled” low-level activation of clotting on the device surface was necessary for endothelialization and that occurred after cessation of Clopidogrel.

### Minor AE events

No serious complications like strokes or transient ischaemic attacks resulted from patients experiencing AF after IASC-C in our population [8], partially because the follow up was tight and the threshold for the indication of vigorous treatment (cardioversion, transient oral anticoagulation and antiarrhythmic medication) was low.

The patient that later on experienced persistent AF and declined Amiodarone and cardioversion because she was asymptomatic, had been screened for AF prior to PFO closure and was found to be in permanent sinus rhythm. She developed the dysrhythmia 4 months after the intervention. Arguably, AF may have been prevented from establishing itself if the advised therapy would have been applied early in the course. If the developement was to be considered as a major AE, the corresponding incidence in this series would rise to 3.7%.

We did not classify the need for CA as a major or intermediate AE in the 69-year-old patient with transient air embolism for the following reasons. Although it is not our practice in asymptomatic low risk patients, most centers would perform routine CA during IASC-C in persons above 65 years of age. Furthermore, CA in our patient did not necessitate a separate cath lab stay nor did it prolong hospitalization or cause extra cost (except for two coronary catheters and a sheath). In the recumbent patient, air embolism originating from the left atrium and ventricle as in our case, mostly involves the right coronary artery, the ostium of which lies anteriorly, preferentially placing it in the path of the less densely aggregated air bubbles, compared to blood. Fortunately, in most patients the take off of the aortic arch branches is directed slightly caudally, so that again gravity influences their course favourably in the recumbent patient. Air bubbles that do reach the coronary or cerebral circulation are happily few and they dissolve rapidly in the circulation. Obviously every effort should be undertaken to avoid even minute amounts of air bubbles by careful flushing of the system prior to loading it. The only system (Solysafe®) not requiring a sheath to be placed across the IASC, rendering air embolization virtually impossible has been withdrawn from the market. On the other hand, the Amplatzer® system does have the advantage that it allows backflow of blood when its end is lowered below heart level, once the sheath has crossed the defect, so that if the introducer and the device have been thoroughly flushed, there is no risk of air embolism, except in the rare case where the patient experiences a severe bout of coughing when the system is open. For other sheath systems, connecting the sheath with a constant saline flush under pressure is another way of preventing air entering through the back-bleed valve.

### Partially successful or unsuccessful interventions, “redos”.

Residual shunting in our cases was mainly the result of device undersizing, which we attribute in turn to the lack of balloon measurement of the stretched defect diameter. Since these AE, we routinely size even small PFOs, and we have never experienced any laceration of the septum, mainly because we stop the careful inflation of the balloon as soon as we see a waist on fluoroscopy, thus avoiding overstretching. Initially we advocated classifying re-intervention for definitive closure of a residual shunt as a major AE, in analogy to classification after PCI or coronary surgery [5] in which target vessel revascularization is a major AE. This has been criticized with the argument that the patient presented with a shunt from the start, and that, at worst, a “status quo ante” had been achieved by the intervention at worst. In fact, although the residual shunt is hopefully smaller than the original defect, it cannot yet be stated that it is harmless in terms of major or minor stroke risk. Whether interventional cardiologists decide to consider re-intervention for shunt closure as a major AE or not should be open for discussion. The use of more than one device applied to the same septum has been described before [9].

Again, unsuccessful closure with a “PFO-device” necessitated repeating the procedure another day. We would be in favour to classify this “redo” as a major AE. The argument has been forwarded that this case could be compared to a PCI in which it is realized that the chosen stent does not fully cover the lesion, being followed by it’s exchange for a longer one.

Although the left atrial disc size of the ASD-device is 32 mm and thus smaller than the 35 mm right atrial disc of the PFO device, stability (Fig. 6B) was attained probably by the larger volume of the interdiscal connection of the ASD device. Although more of a semantic problem, the TEE diagnosis of a “large PFO” had to be revised after balloon sizing in favour of a “fossa ovalis ASD”. Beyond semantics though, the case demonstrates that larger PFOs may need to be closed with ASD devices, especially when a large atrial septal aneurysm imparts large amplitude movements to the whole septum and leads thus to an unstable position to device.

One multifaceted question must be considered, namely whether any of the reported complications is related to a general or device-specific learning curve, and whether there is any hint of a device-specific complication. None of the complications seem to be linked to the handling aspect of a learning curve, except the residual shunt with the Premere® device, in which it may be possible that the discs were not pulled together tightly enough. As already mentioned, the residual shunt with the Amplatzer® device and the dislodged device were partly due to the fact that we did not routinely balloon-size PFOs at the beginning of our experience, so that we postulate undersizing in these two cases. It is noteworthy that many centers do not balloon-size PFOs. The patient that experienced tamponade was the 64th in the series, so that a learning curve effect seems improbable. The same reasoning pertains to the patient with an AV fistula. The recurrent thrombus on the Atriasept Cardia® device may or may not be a device specific problem. Dual platelet inhibition and at times anticoagulation was prescribed in a standard manner, a learning curve effect is improbable. Recurrent low grade infection may have contributed to a disturbed clotting mechanism, although coagulation disorders had been ruled out as described. We feel that the number of devices used precludes a statement connecting a specific device to a specific complication.

The question has been raised as to why a specific device was been preferred to others. Obviously larger defects seem to fare better with the classical device designs, namely the Amplatzer® and its “generic” the Occlutech® (cases 1 and 2 under major AE, and the last of the “redo” cases). We have tried “lean” devices for small defects (case 3 under major AE), specifically sometimes the Premere® for tunnelled PFOs (one of the redo cases).

Comparison of AE according to the proposed classification with some larger studies may be interesting. Re-computing the data of the study by Majunke et al [1] yields a major AE rate (both long and short term) of 2.3% (15/641), assuming that patients lost to follow up did not experience any major AE and excluding pericardiocentesis, minor strokes and heart failure (the duration of additional hospitalization or long term medication, if any, not being available). The adjusted analysis of another large study [2] leads to a major AE rate of 1.7% (9/525), also applying the same criteria. Table 2 summarizes the analysis of both studies.

**Table 2:** Major Adverse Events (AE) After Interatrial Septal Communication Closure Using Our Standardized Criteria, as Recomputed From 2 Large Studies [1, 2]

	Major AE	Not included
Majunke et al [1] Total major AE: 15/641 = 2.3%	Early: **2** embolizations, surgery **1** residual shunting, percutaneous closure unsuccessful, surgery **1** sudden death After 30 days: **2** embolizations and percutaneous retrieval **2** embolizations, surgery **3** surgery for atrial roof erosion **1** major stroke **1** peripheral embolization and percutaneous retrieval **2** surgery for residual shunt	4 Heart failure 3 Minor strokes or TIA 1 pericardiocentesis 1 atrial thrombus formation 10 percutaneous closure for residual shunt or several defects
Wahl et al [2] Total major AE: 9/525 = 1.7%	Early: **5** embolization and percutaneous retrieval **3** vascular access problems **1** retroperitoneal hematoma	2 pericardiocentesis 4 atrial thrombus formation 16 repeated closure because of residual shunt or new defects

Some AE listed on the right hand column are not included in the computing because it is not clear whether they necessitated hospitalizations etc. We have classified other events (residual shunts and “redos”) as unsuccessful or partially successful, in analogy to our 3 cases.

Caution has to be exercised when comparing data though, as it must be taken into consideration that some of the devices used in one study were precursors providing a less favourable profile [2]. On the other hand, the study by Majunke et al [1] describes an experience in ASD closure, which may inherently be more challenging than PFO closure. Both studies in question were multi-operator studies.

**Figure 8:**
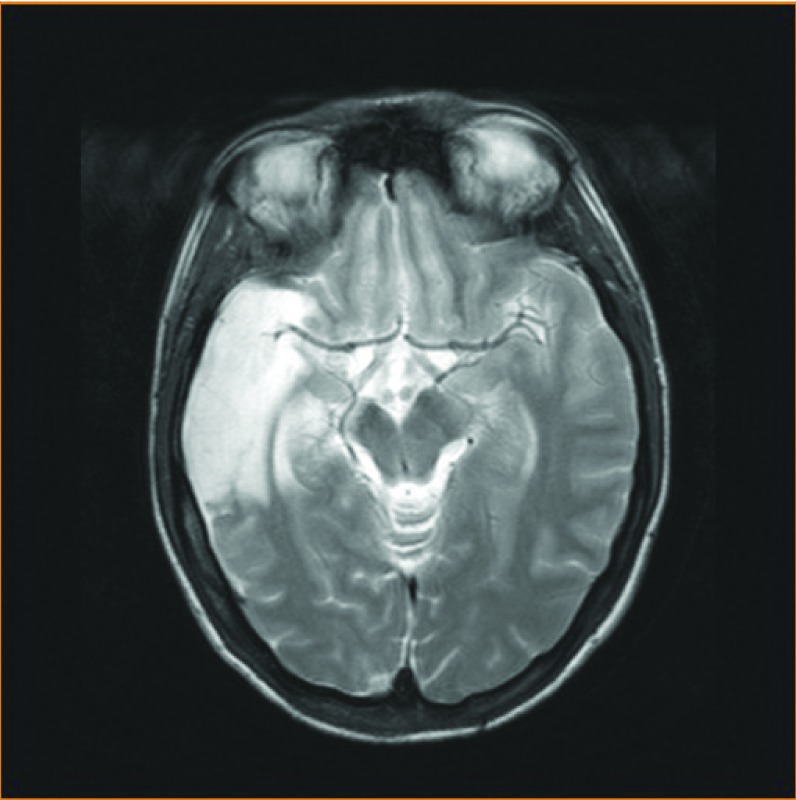
MRI sequence of a severe lesion in the area subtended by the middle cerebral artery. The injury was sustained before PFO closure, most probably due to paradoxical embolism after documented deep venous thrombosis in a 25-year-old woman.

All in all and despite one very severe adverse event, we are still convinced that closure of IASC is warranted provided careful patient selection precedes the intervention. Paradoxical embolism and severe disability (Fig. 8) can hopefully sometimes be prevented by PFO closure, especially in patients who experience a “warning ”cerebrovascular event [7].

### Conclusion

The proposed new classification of AE provides a unified and comparable approach for IASC procedures. As far as we can judge, two of the three major AE may have been avoided by paying more attention to sizing. In one case, balloon sizing was not performed at all, undersizing may have contributed to subacute device embolization. Following this experience, we angiographically “balloon-sized” all IASC. On the other hand, some oversizing undetected by TEE probably contributed to late erosion of the atrial roof and tamponade. Intrainterventional TEE plays an eminent role in yielding precise information about potential encroachment of the device on neighbouring structures, such as the aortic root or the atrial roof, but is, in our experience, less useful as a stand alone sizing method. The use of 3D TEE may prove an additional asset in the future [10]. Last but not least and in the light of our own findings, we advocate a less cavalier approach when it comes to IASC-C, while still being convinced that the indication of this procedure is sound in the majority of cases.
